# High expression of α-synuclein in damaged mitochondria with *PLA2G6* dysfunction

**DOI:** 10.1186/s40478-016-0298-3

**Published:** 2016-03-30

**Authors:** Hisae Sumi-Akamaru, Goichi Beck, Koei Shinzawa, Shinsuke Kato, Yuichi Riku, Mari Yoshida, Harutoshi Fujimura, Yoshihide Tsujimoto, Saburo Sakoda, Hideki Mochizuki

**Affiliations:** Department of Neurology, Graduate School of Medicine, Osaka University, 2-2 Yamadaoka, Suita, 565-0871 Japan; Department of Medical Genetics, Graduate School of Medicine, Osaka University, 2-2 Yamadaoka, Suita, 565-0871 Japan; Division of Neuropathology, Department of Brain and Neurosciences, Tottori University Faculty of Medicine, 86 Nishi-machi, Yonago, 683-8504 Japan; Department of Neurology, Nagoya University Graduate School of Medicine, 65 Tsurumai-cho, Showa-ku, Nagoya 466-8550 Japan; Institute for Medical Science of Aging, Aichi Medical University, 9 Yazakokarimata, Nagakute, 480-1195 Japan; Department of Neurology, National Hospital Organization Toneyama National Hospital, 5-1-1 Toneyama, Toyonaka, 560-8552 Japan; Research Institute Osaka Medical Center for Cancer and Cardiovascular Diseases, 1-3-3 Nakamichi, Osaka, 537-0025 Japan

**Keywords:** PLA2G6, α-synuclein, Mitochondrial membrane, Lewy body

## Abstract

To clarify the role of α-synuclein (αSyn) in neuronal membrane remodeling, we analyzed the expression of αSyn in neurons with a dysfunction of PLA2G6, which is indispensable for membrane remodeling. αSyn/phosphorylated-αSyn (PαSyn) distribution and neurodegeneration were quantitatively estimated in *PLA2G6*-knockout (KO) mice, which demonstrate marked mitochondrial membrane degeneration. We also assessed the relationship between αSyn deposits and mitochondria in brain tissue from patients with PLA2G6-associated neurodegeneration (PLAN) and Parkinson’s disease (PD), and quantitatively examined Lewy bodies (LBs) and neurons. The expression level of αSyn was elevated in *PLA2G6*-knockdown cells and KO mouse neurons. Strong PαSyn expression was observed in neuronal granules in KO mice before onset of motor symptoms. The granules were mitochondrial outer membrane protein (TOM20)-positive. Ultramicroscopy revealed that PαSyn-positive granules were localized to mitochondria with degenerated inner membranes. After symptom onset, TOM20-positive granules were frequently found in ubiquitinated spheroids, where PαSyn expression was low. Axons were atrophic, but the neuronal loss was not evident in KO mice. In PLAN neurons, small PαSyn-positive inclusions with a TOM20-positive edge were frequently observed and clustered into LBs. The surfaces of most LBs were TOM20-positive in PLAN and TOM20-negative in PD brains. The high proportion of LB-bearing neurons and the preserved neuronal number in PLAN suggested long-term survival of LB-bearing neurons. Elevated expression of αSyn/PαSyn in mitochondria appears to be the early response to PLA2G6-deficiency in neurons. The strong affinity of αSyn for damaged mitochondrial membranes may promote membrane stabilization of mitochondria and neuronal survival in neurons.

## Introduction

It is well known that α-synuclein (αSyn) is a pathological marker of Parkinson disease (PD), because αSyn/phosphorylated αSyn (PαSyn) is a main component of Lewy bodies (LBs). The physiological function of αSyn has recently become known. As a pre-synaptic chaperone, αSyn promotes soluble NSF-attachment protein receptor (SNARE)-complex assembly [9, 22]. αSyn also localizes to nuclei and subcellular organelles, including mitochondria and mitochondrion-associated endoplasmic reticulum (ER) membranes [5, 16, 37]. αSyn binds to lipid membranes [42, 43], in particular, to membranes with high curvature, such as synaptic vesicles and mitochondrial inner membranes [5, 25, 52]. The N-terminus of αSyn lies along the surface of the membrane [5, 43], where it senses lipid-packing defects and leads to membrane remodeling and stabilization [7, 29].

Mitochondria comprise an inner and an outer membrane that separate the intermembrane space and the matrix. Mitochondria have various functions, including oxidative phosphorylation, lipid metabolism, endocytosis [24], apoptosis, and calcium and iron homeostasis [32]. A mitochondrial inner membrane-specific phospholipid, cardiolipin, is crucial for the integrity and function of mitochondria [25, 32]. In the brains of mice lacking αSyn, the mitochondrial lipid composition changes, and complex I/III activity is reduced [12]. Moreover, the N-terminus of αSyn regulates mitochondrial membrane permeability [40]. Together, these findings suggest that αSyn is integral to maintaining mitochondrial function.

Calcium-independent phospholipase A_2_β, encoded by *PLA2G6*, has diverse functions, such as releasing lipid mediators, inflammation, vascular relaxation, and secretion, by hydrolyzing the *sn*-*2* ester bond in phospholipids [8, 10, 27, 28, 51]. In *PLA2G6*-associated neurodegeneration (PLAN) [19, 26], formerly called Seitelberger disease, a variety of neurological deficits are present from infancy, suggesting the importance of *PLA2G6* in the brain. PLAN encompasses a number of phenotypes, such as infantile neuroaxonal dystrophy and adult-onset dystonia-parkinsonism (Park14). Although these phenotypes differ in the degree and distribution of neurodegeneration, αSyn/Lewy-related pathology is commonly observed [15, 31, 36].

The pathogenesis of *PLA2G6* deficiency is thought to involve dysfunction of mitochondria and membrane remodeling [13, 20]. *Pla2g6*-knockout (KO) mice show slow progression of motor deficits, and there is a progressive formation of spheroids and tubulovesicular structures [23, 41], similar to that seen in PLAN [11, 17]. Recently, we reported that the spinal cord neurons in *Pla2g6*-KO mice have ultra-microscopically abnormal mitochondria, with degenerated inner membranes, which are periodic acid Schiff (PAS)-positive, negative for an inner membrane protein (cytochrome c oxidase, CCO), and positive for an outer membrane protein (translocase of the outer mitochondrial membrane, TOM20) on immunohistochemistry [4, 45]. In mass spectrometry, the content of mitochondria-specific phospholipid, cardiolipin, is high in *Pla2g6*-KO mice. These findings suggest dysregulation of phospholipid metabolism in the mitochondrial inner membrane. We also previously demonstrated low expression of CCO and low production of ATP in *PLA2G6*-knockdown cells, suggesting mitochondrial dysfunction [3].

Genetic mutation of *SNCA* [34] and multiple copies of *SNCA* [38] cause PD, suggesting the toxic function due to a genetic abnormality of αSyn [5, 48]. On the other hand, in the brains of elderly people without neuropsychiatric symptoms, where widespread αSyn/Lewy-related pathology is seen [39], the severity of αSyn/Lewy-related pathology is not associated with the clinical course in PD [14, 18]. In spite of many intensive researches [1, 5, 14, 18, 35, 48], the biological significance of LBs in sporadic PD and other familial PD is not yet fully understood. In this study, we aimed to clarify the reason for αSyn accumulation in neurons, and pathologically analyzed the relationship between αSyn and mitochondrial membranes in PLAN and in *Pla2g6*-KO mice.

## Materials and methods

### Generation of Pla2g6-Kd cells

We generated *PLA2G6* gene knockdown (Kd) SH-SY5Y human neuroblastoma cells, as described before [3]. Briefly, SH-SY5Y neuroblastoma cell line was obtained from American Tissue Culture Collection (ATCC, Manassas, VA). Cells were grown in Dulbecco’s modified Eagle’s medium high glucose (high-glucose formulation, Nacalai Tesque, Kyoto, Japan) supplemented with 10 % fetal bovine serum, 100 units/ml penicillin, and 100 μg/ml streptomycin. Cell cultures were all kept at 37 °C. The small interfering RNA (siRNA) targeting human *PLA2G6* gene (Life technologies, Carlsbad, CA) and negative control siRNA (Qiagen, Hilden, Germany), were obtained. Subconfluent SH-SY5Y cells were transfected with siRNAs using Lipofectamine RNAiMax (Invitrogen, Carlsbad, CA). The targeting sense sequence for human *PLA2G6* in SH-SY5Y cells is 5′-GACCAAAGAGCAAGUGACAAAUGUU-3′.

### RNA expression analysis

The absence of the *PLA2G6* expression was confirmed in *Pla2g6*-Kd cells, as described before [3]. Briefly, total RNA was extracted from siRNA-transfected SH-SY5Y cells using the RNeasy Kit (Qiagen, Hilden, Germany), and the RNA concentrations were determined spectrophotometrically. cDNA was generated using the SuperScript VILO cDNA Synthesis Kit (Invitrogen, Carlsbad, CA) from 100 ng of each RNA sample. RT-PCR was used to confirm reduced expression levels of *PLA2G6* gene (data not shown).

### Western blotting

Cells were collected after transfection for 48 h. Samples (*n* = 6 per group) were prepared as described before [3]. Cells, which were transfected with negative control siRNA, were used as control. The protein (10 μg) was separated on 15 % SDS-PAGE, electrotransferred to a polyvinylidene difluoride (PVDF) membrane (Bio-Rad, CA, USA), blocked with 5 % nonfat milk and incubated overnight at 4 °C with the primary antibody against GAPDH (1:1000, Millipore) and α synuclein (αSyn, 1:200, IBL, Fujioka, Japan). The bands were visualized with enhanced chemiluminescence’s reagents and exposed to X-ray film.

### Immunocytochemistry

Cells were fixed in 4 % paraformaldehyde for 30 min. After washing with PBS three times, cells were permeabilized with 0.2 % Triton X-100 for 30 min, and incubated with 10 % skim milk in PBS for 60 min. The antibodies against 20-kDa translocase of the outer mitochondrial membrane (1:100 dilution, TOM20, import receptor, expressed on the mitochondrial outer membrane; Santa Cruz, Dallas, TX) and αSyn (1:100 dilution, LB509, Abcam, Cambridge, England) were used as primary antibodies. Alexa Fluor1488 goat anti-rabbit IgG (H + L) antibody (Life Technologies) and Alexa Fluor1568 goat anti-mouse IgG (H + L) antibody (Life Technologies) were used as the secondary antibodies. Confocal laser-scanned images were obtained using an LSM 510 META (Carl Zeiss, Oberkochen, Germany).

### Animals

All animals were handled in accordance with the Guidelines for Animal Experimentation of Osaka University (No.26-044-000).

Mice carried a homozygous disruption of *Pla2g6* on a C57BL/6 background [41], aged 15 weeks (*n* = 3, pre-clinical stage, 1 male and 2 females); 1 year (*n* = 4, early symptomatic stage, 1 male and 3 females); and 2 years (*n* = 5, end stage, 2 males and 3 females); and wild-type mice, aged 15 weeks (*n* = 3, 1 male and 2 females); and 2 years (*n* = 4, 1 male and 3 females) were obtained. After perfusion of 4 % paraformaldehyde, spinal cords were removed and immersed in the same fixative overnight at 4 °C, after which 4-μm–thick paraffin sections were prepared. Sciatic nerves were also obtained. The sciatic nerves and small pieces of the spinal cord were fixed with 2.5 % glutaraldehyde and processed to epon blocks, as described previously [44]. Epon sections (1-μm–thick) were stained with thionine and PAS.

### Autopsy

The research presented in this study has been approved by the University Ethics Committee Osaka University Graduate School of Medicine (No. 10038). Consent for autopsies were obtained from legal representatives for all subjects in accordance with local institutional review board requirements, which was approved by the University Ethics Committee (Osaka University Graduate School of Medicine, Osaka, Japan).

Autopsy samples of one PLAN case (age at death: 20 years; disease duration: 17 years), seven Parkinson’s disease cases (age at death: 73 ± 9 years; disease duration: 13 ± 8 years), and five non-neurodegenerative control cases (age at death: 61 ± 16 years) were obtained. Paraffin-embedded 6-μm–thick sections of the midbrain, at the level of the red nucleus, upper pons, and upper medulla, were prepared and examined, because of the high frequency of LBs in those area in both PLAN [15, 31, 36] and PD.

### Immunohistochemistry

Deparaffinized sections were incubated for 30 min with 0.3 % H_2_O_2_ to quench endogenous peroxidase activity, and were then washed with phosphate-buffered saline (PBS). The primary antibodies used were rabbit polyclonal antibodies against α-Syn (1:2000 dilution for mouse, 1:5000 dilution for human; Sigma − Aldrich, St. Louis, MO), TOM20 (1:40 dilution for mouse, 1:100 dilution for human, Santa Cruz), cathepsin D (lysosomal enzyme, 1:400 dilution, Dako, Glostrup, Denmark), ubiquitin (1:2000 dilution, DAKO), and mouse monoclonal antibodies against PαSyn (specific for Ser129-phosphortlated αSyn, 1:800 dilution for mouse, 1:2000 dilution for human, pSyn#64; Wako, Osaka, Japan) and cytochrome *c* oxidase subunit IV (CCO, respiratory complex IV, expressed on the mitochondrial inner membrane; 1:300 dilution for mouse, 1: 1000 dilution for human; Invitrogen) and KDEL (Lys-Asp-Glu-Leu, 1:500 dilution, Enzo Life Sciences, Farmingdale, NY). Goat anti-rabbit and anti-mouse immunoglobulins conjugated to peroxidase-labeled dextran polymer (Dako Envision+, Dako) were used as secondary antibodies. Reaction products were visualized with 3,3′-diaminobenzidine tetrahydrochloride (Vector Laboratories, Burlingame, CA), and hematoxylin was used to counterstain the cell nuclei. The immunostaining patterns were compared in serial sections. Some sections were additionally stained with Luxol Fast Blue (LFB) or PAS.

### Double immunohistochemistry

For double immunohistochemistry, two primary antibodies were combined, including antibodies for αSyn (αSyn or PαSyn), mitochondrial membrane markers (CCO or TOM20), ubiquitin, and tyrosine hydroxylase (TH). The VECTASTAIN ABC-AP kit (Vector Laboratories) and ALKALINE PHOSPHATASE SUBSTRATE KIT IV BCIP/NBT (Vector Laboratories) were used for the secondary antibody and visualization of reaction products, respectively.

### Quantitative pathological analysis of anterior horn cells and sciatic nerves of mice

We estimated the number of neurons filled with PαSyn-positive granules and the number of motor neurons in the anterior part of mouse cervical spinal cord and myelinated fibers in sciatic nerves. The neurons with clear nucleoli and cell bodies with a diameter greater than 25 μm, presumed to be alpha motor neurons, were counted [44]. To this end, video images were obtained for each 4-μm–thick Nissl-stained paraffin section and each 1-μm–thick toluidine blue-stained epon section, using a digital camera (KEYENCE VB-7010, KEYENCE, Osaka, Japan) attached to a light microscope (ECLIPSEE800, Nikon, Tokyo, Japan). The diameters of motoneurons showing clear nucleoli and cell bodies, and the myelinated fibers in the sciatic nerves were measured using image analysis software (VH-H1A5, KEYENCE). Four sections of cervical cords were examined for each mouse. For the sciatic nerves, three fields (100× magnification) per mouse were examined. The number of motoneurons, large myelinated fibers (diameter, >10 μm), and total myelinated fibers in wild-type mice (2-years-old) and *Pla2g6*-KO mice (1-year-old and 2-years-old) was counted. The density of myelinated fibers (per mm^2^) was calculated for each mouse. The differences in the number of motoneurons and the fiber density between wild-type mice and *Pla2g6*-KO mice were statistically analyzed using the Wilcoxon rank sum test.

### Ultrastructural analysis

Ultrathin sections of the spinal cord from *Pla2G6*-KO mice aged 15 weeks, 56 weeks, or 100 weeks were prepared and stained with uranyl acetate and lead citrate, and examined using a transmission electron microscope (H-7650; Hitachi High-Technologies Co., Tokyo, Japan).

### Quantitative pathological analysis of TOM20-expression on the surface of human LBs

Double immunohistochemistry was performed for PαSyn (in brown) and for TOM20 (in blue) in the substantia nigra, in the locus coeruleus, or in the dorsal motor nuclei of the vagus nerve. Neuronal round or spherical PαSyn-positive inclusions with a clear contour were defined as LBs. In double immunohistochemistry, PαSyn-positive LBs (brown) were separated into three types, according to the expression pattern of TOM20 (blue) on the surface: TOM20-negative, partially TOM20-positive, and completely TOM20-positive (Fig. 7). Where less than half of the LB surface was positive for TOM20, the LB was classified as “partially TOM20-positive” and where more than half of the LB surface was positive for TOM20, the LB was classified as “completely TOM20-positive”. We counted the number of each type of LB in the substantia nigra, the locus coeruleus, and dorsal motor nuclei of the vagus in each patient. Slides were examined with a × 40 objective, and the microscope images were viewed on video-screen. The contours of the substantia nigra and the locus coeruleus, and dorsal motor nuclei of the vagus were drawn on the slide. The number of LBs with various surface expression levels of TOM20 was compared between PLAN and PD sections.

### Estimation of dopaminergic neuron density and the proportion of LB-bearing neurons in controls and PD and PLAN patients

Double immunohistochemistry was performed against PαSyn (brown) and TH (blue) in human brainstem sections. In double immunohistochemistry, we counted the number of TH-positive (dopaminergic) neurons bearing LBs and total TH-positive neurons in the substantia nigra and the locus coeruleus [47]. Slides were examined with a × 40 objective, and the microscope image was viewed on a video-screen. The contours of the substantia nigra and the locus coeruleus were drawn on the slide. The neuron density over three fields was calculated for each patient. The density (per mm^2^) of TH-positive neurons bearing LBs and total TH-positive neurons were compared between PLAN, PD (*n* = 7), and control (*n* = 5) individuals.

### Estimation of the influence of LBs on TH expression levels in dopaminergic neurons

Double immunohistochemistry was performed for PαSyn (brown) and TH (blue). The immunostaining of TH was compared between dopaminergic neurons with LBs and those without LBs, in the substantia nigra and locus coeruleus. The brainstem was compared between PLAN and PD (*n* = 7) individuals.

### Statistical analysis

All statistical analyses of histopathological data in mice were performed using SPSS Statistics software for Windows version 20.0 (SPSS, Chicago, IL). Data are expressed as the mean (SD). Mann–Whitney *U* test was used to analyze the ratio of αSyn/GADPH in cultured cells, the number of neurons and the density of large myelinated fibers and total myelinated fibers in mice. *P* values of less than 0.05 were considered as statistically significant.

## Results

### Cultured cells

#### High expression of αSyn in Pla2g6-knockdown cells

**Fig. 1:**
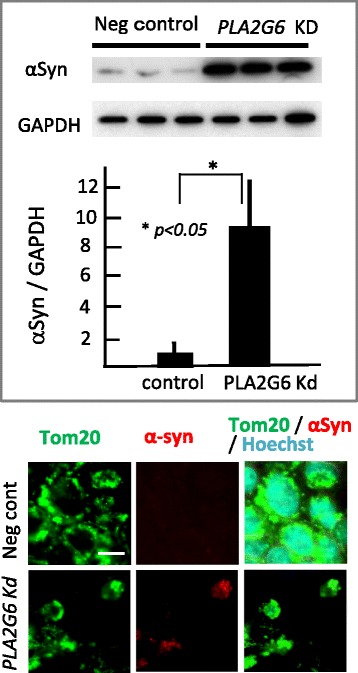
Elevated expression of αSyn in *Pla2g6*-knockdown (Kd) SH-SY5Y cells. Western blotting analysis (upper panel) and the immunocytochemistry (lower panel). Normalizing by GAPDH, the expression level of a-syn in PLA2G6 Kd cells was estimated (*n* = 6 per group). Each bar represents the mean ± SD. *p < 0.05. Mitochondria or nucleus was visualized by a-Tom20 antibody and Hoechst33342 staining, respectively. Scale bar represents 10 μm

To clarify the relationship between αSyn and PLA2G6 dysfunction in cultured neurons, we analyzed the expression level of αSyn in *Pla2g6*-Kd SH-SY5Y human neuroblastoma cells. In the western blotting analysis of *Pla2g6*-Kd cells, αSyn expression was strikingly high (Fig. 1). The difference between Kd cells and negative control cells was significant (*n* = 6 per group, *p* < 0.05). Colocalization of αSyn and translocator of outer mitochondrial membrane 20 (TOM20) was demonstrated by immunocytochemistry.

### Mice

#### Strong expression of αSyn in the spinal cord of Pla2g6-KO mice at the preclinical-stage

**Fig. 2:**
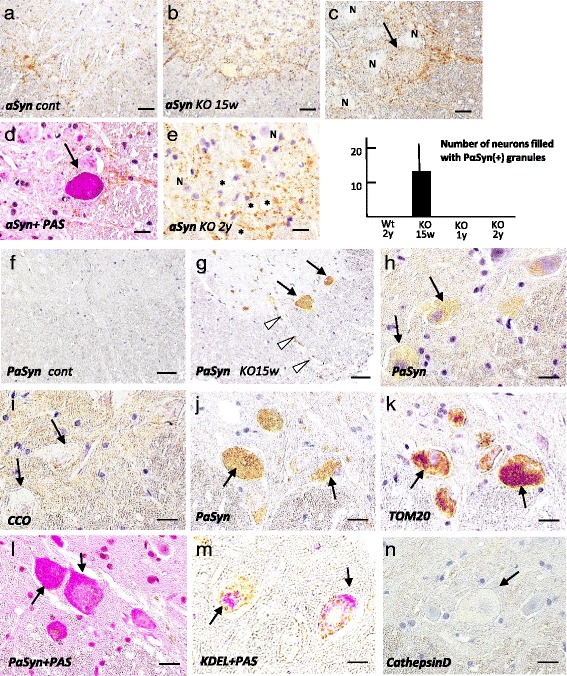
Prominent accumulation ofαSyn/phosphorylated αSyn (PαSyn) in the spinal cord of *Pla2g6*-knockout (KO) mice at the preclinical-stage. **a**, **f** wild-type mice; **b**-**d**, **g**-**m**, *Pla2g6*-KO mice at 15 weeks; **e**, *Pla2g6*-KO mice at 2 years; **a**-**n**, Anterior part of spinal cords; **a**-**c**, **e**, Immunohistochemistry for αSyn; **d**, Immunohistochemistry for αSyn plus Periodic Acid Shiff (PAS); **f**-**n**, Immunohistochemistry against PαSyn (**f**-**h**, **j**), CCO (**i**), and TOM20 (**k**), and cathepsin D (**n**), Immunohistochemistry for PαSyn (l) or KDEL (**m**) plus PAS, respectively; Scale bar represents 80 μm in (**a**, **b**, **f**, **g**), and 20 μm in (**c**-**e**, **h**-**n**). The graph shows the number of neurons filled with PαSyn-positive granules in wild-type mice at 2 years of and *Pla2g6*-KO mice at 15 weeks, 1 year, and 2 years of age. **a** The gray matter of the control spinal cord is moderately stained, showing a punctate pattern; **b**, **c** αSyn expression is highly diffuse in the gray matter of young *Pla2g6*-KO mice. The neuron is mildly positive for αSyn (arrow in **c**), which is filled with PAS-positive granules, as shown in a serial section (**d**). **e** In the gray matter of KO mice at end stage, several vacuoles and spheroids are seen, which are negative for αSyn (*). **f** No staining is seen in the control. (**g**) In KO mice at the pre-clinical stage, there are many tiny PαSyn-positive granules in the anterior horn cells (*arrows*) and the proximal axons (white arrowheads). **h**, **i**, PαSyn-positive small granules (arrows in h) are virtually negative for CCO (arrows in **i**) in the same neurons as in the serial section. **j**, **k** Very small PαSyn-positive granules (arrows in **j**) are prominently observed in the same neurons filled with TOM20-positive granules (arrows in **k**) in the serial section. **l**, **m**, PAS-positive granules are PαSyn-positive (**l**, arrows) and KDEL-negative (**m**, *arrows*). **n** A swollen neuron is negative for cathepsin D (*arrow*)

To clarify the relationship between αSyn and PLA2G6 dysfunction in mice in vivo, we analyzed the distribution of αSyn in *Pla2g6*-KO mice using immunohistochemistry. In wild-type mice of both 15 weeks and 2 years of age, the neuropil was moderately stained for αSyn, with punctate pattern (Fig. 2a). In *Pla2g6*-KO mice at the pre-clinical stage (15 weeks), the immunoreactivity of αSyn was highly diffuse in the gray matter (Fig. 2b). αSyn staining in the neuropil had a punctate pattern, similar to that in wild-type mice, but the cytoplasm of some swollen neurons was also mildly positive for αSyn (Fig. 2c). PAS staining showed that αSyn-positive swollen neurons were filled with PAS-positive granules (Fig. 2d). With age, large vacuoles and spheroids in the neuropil were increased in number, where the expression level of αSyn was almost none or low (Fig. 2e).

#### Strong expression of PαSyn in CCO-negative, TOM20-positive granules in young Pla2g6-KO mice

To determine if there was a relationship between subcellular micro-organelle localization and accumulation of PαSyn, the most important modified-form of αSyn after transcription, we compared the distribution of PαSyn with markers of subcellular micro-organelles in neurons of *Pla2g6*-KO mice. Immunohistochemistry with an antibody specifically directed against Ser129-phosphorylated αSyn (pSyn#64) revealed the absence of PαSyn in the wild-type mice (Fig. 2f). In *Pla2g6*-KO mice at 15 weeks, very small PαSyn-positive granules were prominent in the cytoplasm and proximal axons of anterior horn cells (Fig. 2g, h, j) and dorsal root ganglion cells (data not shown). In some of the swollen neurons filled with small PαSyn-positive granules, the nuclei were atrophic (Fig. 2h, j). By comparing serial sections, we found that the neurons filled with PαSyn-positive granules were CCO-negative (Fig. 2i) and TOM20-positive (Fig. 2k). The granules were PAS-positive (Fig. 2l), KDEL-negative (Fig. 2m), and cathepsin D-negative (Fig. 2n). The number of neurons filled with PαSyn-positive granules is shown in the graph (Fig. 2) and Table 1.

**Table 1:** The time course of Pα-syn expression in neurons and the number of motor neurons and myelinated fibers of *Pla2g6*-knockout (KO) mice

		Wt		KO	
		2y (*n* = 4)	15w (*n* = 3)	1y (*n* = 4)	2y (*n* = 5)
Number of neurons filled with PαSyn (+) granules		0	12 (10)	0	0
Number of AHCs		44 (6)	n.e.	44 (2)	33 (10)
Sciatic nerve (distal axon)	Density of myelinated fibers	17567 (1513)	n.e.	14281 (2086)	18850 (1745)
Sciatic nerve (distal axon)	Density of large myelinated fibers	1623 (314)	n.e.	613 (172)	156* (104)

*P*α*Syn* phosphorylated α-synuclein, *Wt* wild-type, *KO Pla2g6*-knockout, *15 w* 15 weeks, *1 y* 1 year, *2y* 2 years, *AHC* anterior horn cell, *number of anterior horn cells* mean (SD), *n.e*. not examined, *Density of myelinated fibers* number of myelinated fibers per mm^2^, *large myelinated fibers* myelinated fibers with a diameter larger than 10 μm, *p <0.05

### Morphological changes of PαSyn-positive granules in Pla2g6-KO mice with age

**Fig. 3:**
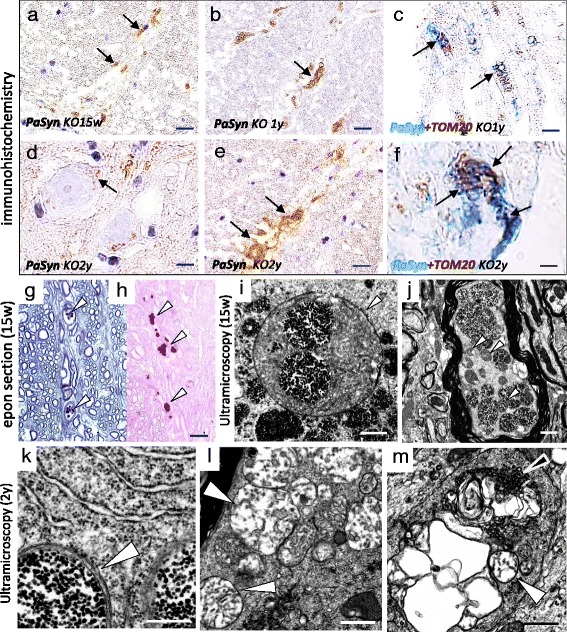
Age-dependent morphological changes in PαSyn-positive mitochondria in axons. **a**-**f** paraffin section; **g**, **h** epon semi-thin section; **i**-**m** ultramicroscopy; **a**, **g**-**j** *Pla2g6*-KO mouse at 15 weeks; (**b**, **c**) *Pla2g6*-KO mice at 1 year; **d**-**f**, **k**-**m**, *Pla2g6*-KO mice at the 2 years. **a**, **b**, **e**, **g**, **h**, **j**, **l**, **m** Anterior column of the spinal cord; **c**, **f** anterior root; **d**, **i**, **k** perinuclear of neuron; **a**, **b**, **d**, **e** Immunohistochemistry for PαSyn; **c**, **f** double immunohistochemistry for PαSyn (*blue*) and TOM20 (*brown*); **g** thionine staining; **h** PAS staining. Scale bars represent 40 μm in (**a**, **b**, **e**), 10 μm in (**c**, **d**, **g**, **h**), 5 μm in (**f**), 500 nm in (**i** and **k**), and 1 μm in (**j**, **l**, **m**). **a** At preclinical-stage, small PαSyn-positive granules (*arrows*) could be seen in axons. **b** At early symptomatic stage, the PαSyn-positive granules became large and irregular-shaped (*arrows*). **c** Co-localization of PαSyn and TOM20 is evident (*arrows*) in the granules. **d** At end stage, PαSyn-positive granules and membranes (*arrow*) are seen in neurons and in the neuropil. **e** PαSyn-positive membranous structures (*arrows*) coincide with the small PαSyn-positive granules. **f** The membranous structures and granules that are positive for PαSyn and TOM20 combine into a complex (*arrows*). **g**, **h** In the axons, there are many dark-colored granules (*arrows* in **g**), which are clearly stained by PAS (*arrow* in **h**). **i** In the perinuclear space of swollen neuron filled with granules, mitochondrion with partly degenerated crista (*arrowhead*) and numerous round bodies filled with dense granules. **j** In a myelinated axon, many spherical mitochondria and round bodies filled with dense granules are seen. The structure of the axon is virtually normal. **k** In the mitochondrion (*arrowhead*) around the nuclei of neurons, most of the cristae are replaced by dense granules. **l** Many mitochondria with tubular and vesicular cristae (*arrowheads*) cluster in the axon. **m** Membranous degeneration, tubulovesicular structures (*black arrowhead*) and abnormal mitochondria (*arrowhead*) can be seen together in the spheroid

To assess the relationships between high PαSyn expression and neurodegeneration in Pla2g6-KO mice, we analyzed the age-dependent distribution of PαSyn. In *Pla2g6*-KO mice at 15 weeks, very small PαSyn-positive granules were prominently observed in the proximal axons (Fig. 3a) and cytoplasm of anterior horn cells. At the early symptomatic stage (1 year), there were only a few PαSyn-positive granules in neuronal cytoplasm. Swollen neurons, filled with PαSyn-positive granules, were completely absent (Table 1). In the proximal axons, there were many PαSyn-positive granules, some of which were large and deformed (Fig. 3b). Double immunohistochemistry revealed PαSyn co-localization with TOM20 in the granules (Fig. 3c).

At the end stage (2 years), some of the PαSyn-positive granules showed degenerated membranes (Fig. 3d, e). Using double immunohistochemistry, PαSyn was shown to co-localize with TOM20 in the granules and the degenerated membrane of the granules (Fig. 3f).

### Semi-thin and ultra-microscopic image of small PαSyn-positive granules in Pla2g6-KO mice

To define the structure of small PαSyn-positive granules in Pla2g6-KO mouse neurons, we compared the the structures observed on semi-thin or ultra-microscopic pictures with the small PαSyn-positive granules seen on paraffin sections. In thionine-stained semi-thin sections, we observed dark colored granules in the cytoplasm and proximal axons of neurons in young KO mice (Fig. 3g). The granules were clearly PAS-positive (Fig. 3h), and showed a similar size and distribution as PαSyn-positive granules. Ultra-microscopically, round mitochondria could be seen in which the cristae were partially replaced by small dense granules both in the perinuclear space of neurons (Fig. 3i) and in axons (Fig. 3j). These abnormal mitochondria and round bodies were of a similar size and had a similar distribution as the PAS-positive granules in semi-thin sections, suggesting that PAS-positive granules were indicative of PαSyn-loading abnormal mitochondria.

In the aged KO mice, fewer round mitochondria could be seen in which the cristae were partially replaced by small dense granules in the perinuclear space of neurons (Fig. 3k). In the axons, mitochondria with branching and tubular cristae were frequently seen, and were clustered together (Fig. 3l). In the spheroid, highly degenerated mitochondria, tubulovesicular structures and degenerated membranes were combined to form an irregular structure (Fig. 3m), suggesting severe degeneration of mitochondrial membranes.

### Different distribution of ubiquitin and PαSyn in Pla2g6-KO mice

**Fig. 4:**
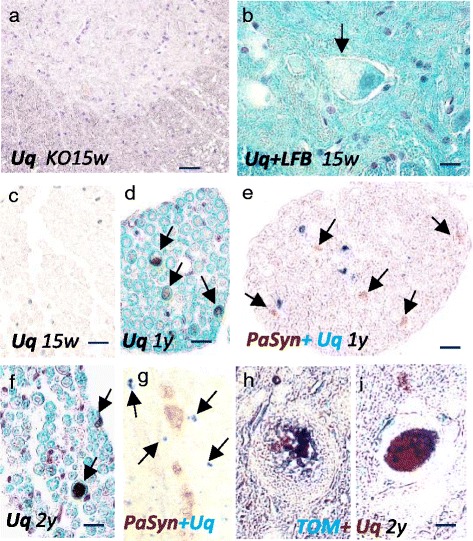
Ubiquitination of mitochondrial membranes at later stages. **a**-**c** *Pla2g6*-KO mouse (15 w); **d**, **e** *Pla2g6*-KO mouse (1 year); **f**-**i** *Pla2g6*-KO mouse (2 years); **a**, **b**, **g** anterior part of the spinal cord; **c**-**f** anterior root; **h**, **i** posterior part of the spinal cord; **a**, **c** immunohistochemistry for ubiquitin; **b**, **d**, **f** immunohistochemistry for ubiquitin plus LFB staining; **e**, **g** double immunohistochemistry for PαSyn (*brown*) and ubiquitin (*blue*); **h**, **i** double immunohistochemistry for ubiquitin (*brown*) and TOM20 (*blue*); Scale bars represent 80 μm in (**a**), 20 μm in (**b**-**g**) and 10 μm in (**h**, **i**). **a** At the pre-clinical stage, there is almost no expression of ubiquitin. **b** The anterior horn cell (*arrow*), filled with small granules, is negative for ubiquitin. **c** In the anterior root, no ubiquitin-positive axon can be observed. **d** At the early symptomatic stage, ubiquitin-positive axons (*arrows*) with LFB-positive (*blue*) myelin are seen in the anterior root. **e** The distribution of small PαSyn-positive granules (*brown*, *arrows*) and ubiquitin (*blue*) are different. **f** Ubiquitin-positive axons (*arrows*) are shown. Most of the myelinated fibers have become atrophic. **g** The distribution of small PαSyn-positive granules (*brown*) and ubiquitin (*blue*) differs. **h** In the center of a large spheroid, TOM20-positive (*blue*) granules are ubiquitin-positive (*brown*, *arrow*). **i** In the markedly ubiquitinated spheroid (*brown*), the expression of TOM20 (*blue*) is still observed

To determine whether the ubiquitin-proteasome system is activated in PαSyn-loading mitochondria, we estimated the relationship between ubiquitin and mitochondrial membrane protein by double immunohistochemistry. At the pre-clinical stage, there were rare ubiquitin deposits in the spinal cord of *Pla2g6*-KO mice (Fig. 4a-c). At the early symptomatic stage (1-year-old), ubiquitin-positive structures were frequently observed in the neuropil and the axons (Fig. 4d), but not in the neuronal cytoplasm. Double immunohistochemistry revealed that the distribution of PαSyn and ubiquitin in the axons was markedly different (Fig. 4e). At the end stage (2-year-old), ubiquitination became severe in axons, but no ubiquitination was observed in the cytoplasm (data not shown). Double immunohistochemistry showed almost no colocalization of PαSyn and ubiquitin (Fig. 4g) but showed frequent colocalization of TOM20 and ubiquitin in the spheroids (Fig. 4h, i).

### Significant decrease in the density of large myelinated fibers, but not neuron number, in the spinal cord

To clarify the degree of neurodegeneration in axons and neurons that contained PαSyn-positive granules, from young mice, we estimated the number of axons and neurons in Pla2g6-KO mice after symptom onset. Axonal degeneration progressed as reported previously [41] and myelinated fibers became atrophic with age. Quantitative analysis (Table 1) revealed a significant decrease in large myelinated fibers (>10 μm in diameter) with age (KO mice 1y, *n* = 4, *p* =0.057; KO mice 2 y, *n* = 5, *p* <0.05). The density of the total myelinated fibers in *Pla2g6*-KO mice after onset was similar to that in the wild-type mice. The number of anterior horn cells in *Pla2g6*-KO mice at the early symptomatic stage was similar to that of wild-type mice. Even at the end stage, the number of neurons was not significantly different between *Pla2g6*-KO and wild-type mice (*p* = 0.067).

### Human

#### Prominent formation of Lewy Bodies (LBs) in a PLAN patient with a mutation in PLA2G6

**Fig. 5:**
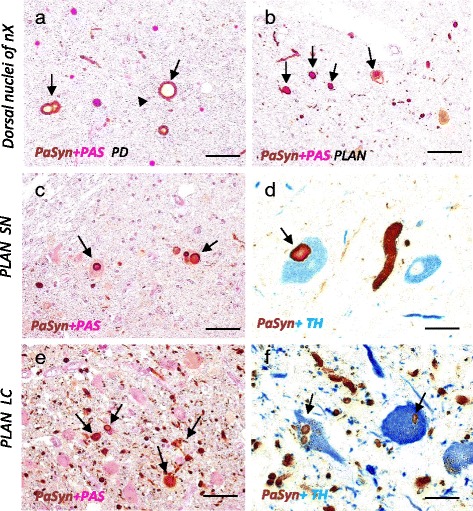
Prominent formation of Lewy bodies in PLAN. **a** Parkinson’s disease (PD) case; **b**-**f** *PLA2G6*-associated neurodegeneration (PLAN) case; **a**, **b** Dorsal motor nuclei of vagus (nX); **c**, **d** Locus coeruleus (LC); **e**, **f** Substantia nigra (SN); **a**-**c**, **e** Immunohistochemistry for PαSyn plus PAS staining; **d**, **f** Double immunohistochemistry for PαSyn (*brown*) and tyrosine hydroxylase (*TH, blue*). Scale bars represent 40 μm in (**a**-**c**, **e**), and 20 μm in (**d**, **f**). **a** In PD, PαSyn-positive inclusions, Lewy bodies (*arrows*) are observed mainly in the neuropil. **b** In PLAN, there are many Lewy neurites and Lewy bodies (*LBs, arrows*) both in the neurons and in the neuropil. **c** Cytoplasmic LBs are indicated with arrows. **d** Spherical Lewy neurites in the center of this panel and a LB in TH-positive neuron (*arrow*) are shown. **e** The neurons in the locus coeruleus are well preserved despite prominent LBs (*arrows*). **f** The LBs in TH-positive neurons are small, but have the apparent core and halo structure (*arrows*)

In the midbrain, upper pons, and upper medulla of both PD and PLAN patients, numerous neuronal inclusions that were positive for αSyn and PαSyn (pSyn#64) could be seen. The affected areas included the substantia nigra, dorsal raphe nucleus, locus coeruleus, dorsal motor nuclei of the vagus nerve, and the reticular formation. Morphologically, the inclusions were round or spherical, similar to Pale bodies or LBs seen in PD (Fig. 5a). In PD, most of the PαSyn-positive LBs were present in the neuropil, particularly in the region showing severe neuronal loss. In the brain of the PLAN patient, LBs were observed both in the neurons and in the neuropil (Fig. 5b-f). The size of LBs in PLAN varied, as it does in PD.

#### PαSyn -positive small inclusions colocalized with the mitochondrial membrane protein in PLAN

**Fig. 6:**
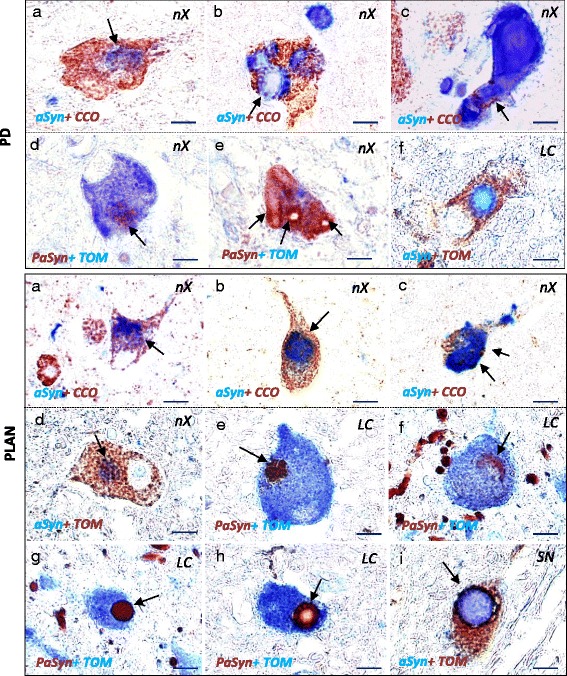
The relationship between the distribution of αSyn or PαSyn and mitochondrial membrane proteins. Upper panels, Parkinson’s disease (PD); **a**-**e** Dorsal motor nuclei of the vagus; **f** Locus coeruleus; **a**-**c** Double immunohistochemistry for αSyn (blue) and CCO (*brown*); **d**, **e** Double immunohistochemistry for PαSyn (*brown*) and TOM20 (*blue*); **f** Double immunohistochemistry for αSyn (*blue*) and TOM20 (*brown*). Lower panels, *PLA2G6*-associated neurodegeneration (PLAN); **a**-**d** Dorsal motor nuclei of vagus; **e**-**h** Locus coeruleus; **i** Substantia nigra; **a**-**c** Double immunohistochemistry for αSyn (*blue*) and CCO (*brown*); **d**, **i** Double immunohistochemistry for αSyn (*blue*) and TOM20 (*brown*); **e**-**h** Double immunohistochemistry for PαSyn (*brown*) and TOM20 (*blue*). Scale bar represents 8 μm in the upper panel and (**a**-**f**) in the lower panel (**a**-**i**). Upper panel, Parkinson’s disease (PD). **a** There is an amorphous deposit of αSyn (*arrow*) in the CCO-positive cytoplasm. **b** In the center of the αSyn-positive LB (*arrow*), the expression of CCO is negative. **c** The sausage-like Lewy body (LB) has a small rim, which is CCO-positive (*arrow*). **d** There are a few PαSyn-positive granules (*arrow*) that are mildly TOM20-positive on the surface. **e** The neuron is filled with sausage-like LBs *arrows*, which are almost negative for TOM20. **f** In the neuron, the surface of the LB is mildly positive for TOM20. Lower panel, *PLA2G6*-associated neurodegeneration (PLAN) **a** There are some CCO-positive dots around the irregular αSyn-positive deposit (*arrow*). **b** αSyn-positive inclusion (*arrow*) contains some CCO-positive granules. **c** CCO expression is seen in only a part of the surface (*arrows*) of αSyn-positive LB. **d** Under the αSyn deposit, there are clear TOM20-positive dots (*arrow*) in the cytoplasm. **e** In the neuronal cytoplasm, there are several PαSyn-positive granules (*arrow*), on which the surface is completely TOM20-positive. **f** PαSyn-positive granules are clustered to form a thin inclusion (*arrow*), on which the surface is positive for TOM20. **g** PαSyn-positive granules with TOM20-positive rim are clustered to form one round inclusion (*arrow*). **h** In the core of the LB, the immunoreaction of PαSyn and TOM20 are absent. In the halo of LB (*arrow*), PαSyn-positive granules with a TOM20-positive rim make a line. **i** PαSyn and TOM20-positive membrane-like structure covers the surface of LB (*arrow*)

To assess the relationship between αSyn/PαSyn deposits and mitochondria, we analyzed the neurons affected in PD and in PLAN using a double immunohistochemical assay. Double immunohistochemistry revealed amorphous deposits of αSyn in the CCO- and TOM20-positive cytoplasm of neurons both in PD and in PLAN patients (PD; Fig. 6a, PLAN; Fig. 6d). As more αSyn accumulated in the neuronal cytoplasm, the CCO-immunoreactivity diminished (PD: Fig. 6b, c, PLAN: Fig. 6a-c). In PLAN neurons, small PαSyn-positive inclusions with a TOM20-positive edge were frequently found, but similar inclusions were rare in PD (PD: Fig. 6d-f, PLAN: Fig. 6e).

### Clustering of PαSyn-positive small inclusions to form a larger neuronal inclusion in PLAN

To clarify the relationship between PαSyn-positive small inclusions and LBs, we analyzed the distribution of PαSyn-positive small inclusions in neurons affected in PLAN. In PLAN neurons, small PαSyn-positive inclusions with a TOM20-positive edge appeared to cluster, and formed a larger narrow (PLAN: Fig. 6f) or round (PLAN: Fig. 6g) inclusion. In some LBs, small PαSyn-positive inclusions with a TOM20-positive edge appeared to cover the surface (PLAN: Fig. 6h), whereas the immunoreactivity of PαSyn was reduced in the center. PαSyn and TOM20-positive membranous structures also appeared to cover the surface of LBs (Fig. 6i).

### Quantitative analysis of LBs with TOM20 present on their surface

**Fig. 7:**
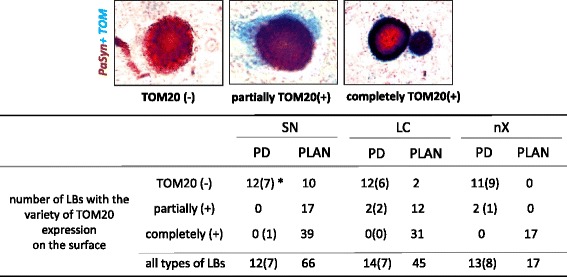
Strong expression of a mitochondrial outer membrane protein (TOM20) on the surface of Lewy bodies (LBs) in *PLA2G6*-associated neurodegeneration (PLAN). The left and the right images show the dorsal nuclei of the vagus in a patient with PLAN. The center image shows the substantia nigra in a patient with Parkinson disease (PD). *TOM20* 20-kDa translocase of the outer mitochondrial membrane, *SN* substantia nigra, *LC* locus coeruleus, *nX* the dorsal nuclei of vagus, *PD* Parkinson disease, *PLAN* PLA2G6-associated neurodegeneration, *LBs* Lewy bodies, *TOM20* (−) TOM20-negative, *partially* (+) partially TOM20-positive, *completely* (+) completely TOM20-positive, *number of LBs* * mean (SD)

To evaluate the relationship between LBs and mitochondria quantitatively, we counted the number of LBs with mitochondrial membrane protein (TOM20) present on their surface in PD and in PLAN. The number of LBs in each area is shown in Fig. 7. In all three investigated areas of the PD brains, most LBs were TOM20-negative on the surface. In the PLAN brain, most LBs were TOM20-positive on the surface. In all three investigated areas of the PLAN brain, more than half of the LBs were completely TOM20-positive. The surfaces of most LBs were TOM20-positive in PLAN and TOM20-negative in PD brains.

### Proportion of LB-bearing dopaminergic neurons and neuronal density

To assess the survival rates of LB-bearing dopaminergic neurons, we estimated the neuronal density and the proportion of LB-harboring neurons in both PD and PLAN. The density of LB-bearing TH-positive neurons and total number of TH-positive neurons in substantia nigra and locus coeruleus are shown in Table 2. In the PD brains, the density of LB-bearing TH-positive neurons was low, because most LBs were found in the neuropil; this density was 1 ± 1 (mean ± S.D.) in the substantia nigra and 2 ± 2 in the locus coeruleus (number per 1 mm^2^). In the PLAN brain, the density of LB-bearing neurons was 6 in the substantia nigra and 17 in the locus coeruleus, per 1 mm^2^. The density of TH-positive neurons in the substantia nigra was low, but that in the locus coeruleus was similar to that in the control brains, despite the prominent presence of LBs (Fig. 5e, f). The proportion of LB-bearing dopaminergic neurons was 6/17 in the substantia nigra and 17/81 in the locus coeruleus, respectively. Thus, a high proportion of LB-bearing neurons and preserved neuronal number were observed in PLAN.

**Table 2:** The dopaminergic neuron density and the proportion of Lewy body-bearing neurons

Area		Density of dopaminergic neurons		
		Control	PD	PLAN
		(*n* = 5)	(*n* = 7)	(*n* = 1)
Substantia nigra	LB harbouring neuron	0	1 (1)	6
	total neuron	84 (14)	13 (6)	17
Locus coeruleus	LB harbouring neuron	0	1 (3)	17
	total neuron	77 (13)	9 (10)	81

*TH* tyrosine hydroxylase, *Density of TH* (+) *neurons* mean (SD) per mm^2^, *control* non-neurodegenerative disease, *PD* Parkinson disease, *PLAN* PLA2G6-associated neurodegeneration, *LB* Lewy body

### Preserved expression of TH in dopaminergic neurons, despite the presence of LBs

To determine if LB influences the micro-environment in dopaminergic neurons, we estimated cytoplasmic TH expression of LB-bearing neurons. Both in PD and PLAN brains, most of the cytoplasmic TH expression in LB bearing neurons was preserved, both in substantia nigra and locus coeruleus (Table 3). Some LB-bearing neurons were TH-negative. No relationship between cytoplasmic TH expression and LB presence was found.

**Table 3:** The expression of tyrosine hydroxylase in dopaminergic neurons bearing Lewy bodies (LBs)

		SN		LC	
		PD	PLAN	PD	PLAN
		(*n* = 7)	(*n* = 1)	(*n* = 7)	(*n* = 1)
Number of LB bearing neurons with the variety of tyrosine hydroxylase expression	TH (+)	3 (2)*	34	2 (4)	19
	TH (−)	1 (1)	3	1 (1)	0
	total number	3 (2)	37	3 (4)	19

*LB* Lewy body, *SN* substantia nigra, *LC*, locus coeruleus, *TH* tyrosine hydroxylase, *TH* (+) TH-positve neuron, *TH* (−) TH-negative neuron, *Number of neurons* mean (SD)

## Discussion and conclusions

In this study, we demonstrated elevated expression of αSyn both in cultured cells and in mice with PLA2G6 deficiency. Prominent accumulation of PαSyn in damaged mitochondria was shown in *Pla2g6*-KO mice, which constitute a model of PLA2G6-associated neurodegeneration (PLAN). In PLAN neurons, small PαSyn-positive granules and Lewy Bodies (LBs) were covered with mitochondrial membrane component on the surface. These findings suggest that αSyn/PαSyn associates with damaged mitochondria in PLAN.

Strong expression of αSyn was evident in *Pla2g6*-Kd neuroblastoma cells and KO mouse neurons, suggesting that endogenous αSyn was induced in neurons due to PLA2G6-deficiency. In *Pla2g6*-KO mice, surprisingly high levels of PαSyn expression were observed in neurons; this was never seen in the wild-type mice. PαSyn was detected on the surface of tiny granules, which had a staining pattern that was apparently different from that of the toxic strains of αSyn, including fibrils and ribbons [6, 33]. Immunohistochemical and ultra-microscopic data suggest that PαSyn-positive and PAS-positive granules were abnormal mitochondria with degenerated inner membranes in KO mice, but not lysosomes or endoplasmic reticulum, as has been shown previously [4, 45].

Injury of the inner mitochondrial membrane leads to permeabilization of mitochondria [2, 32, 50]. In KO mice from young, many neuronal mitochondria with degenerated inner membrane were present, but neither autophagic degradation (mitophagy) nor ubiquitination of mitochondria were observed. Mitochondrial membrane permeabilization also induces apoptosis [32, 49]. More than 8 months after the appearance of mitochondria with degenerated inner membranes, due to *PLA2G6* deficiency, neuronal loss was not significant in mice, suggesting that apoptosis did not occur to neurons. TOM20-positive granules were ubiquitin-negative in neurons and ubiquitin-positive in spheroids, suggesting ubiquitination of mitochondria occurs in degenerated axons, but not in neurons.

In aged KO mice, PαSyn-positive granules became more degenerated morphologically, but the PαSyn expression level was maintained. αSyn/PαSyn seems to have a strong affinity for the mitochondrial membrane [37, 52]. However, considering the different localization of PαSyn and ubiquitin in axons, PαSyn seems to disappear from mitochondria before mitochondrial ubiquitination, following membrane permeabilization. The elevated expression of αSyn/PαSyn in mitochondria may be related to membrane stabilization [40].

In the substantia nigra of 26S proteasome depleted mice, neurodegeneration is accompanied by neuronal inclusions similar to Pale bodies [30], the precursors of LB [46]. The inclusions contain mainly abnormal mitochondria [30], suggesting that Pale body or LB might be associated with abnormal mitochondria with bad quality control owing to dysfunction of ubiquitin proteasome system. As previously reported, LB formation is prominent in PLAN [15, 31, 36]. Considering the severity of mitochondrial degeneration in *Pla2g6*-KO mice, the association of Pale bodies or LBs with mitochondria would be strongly expected in PLAN.

Our double immunohistochemical data in PLAN revealed that the surface of PαSyn-positive small inclusions were TOM20-positive and CCO-negative, which is the same expression pattern of granules seen in *Pla2g6*-KO mouse neurons. αSyn/PαSyn appears to have a strong affinity for mitochondrial membrane components both in *Pla2g6*-KO mice and in the PLAN case investigated here. Moreover, the small inclusions appeared to cluster into a large inclusion, possibly due to the strong affinity of αSyn for the mitochondrial membrane.

LB extracts from PD brains have been reported to trigger αSyn pathology and motor deficits in mice and monkeys, which suggests that LBs contain neurotoxic materials [35]. However, the presence of LBs in dopaminergic neurons was not associated with the low cytoplasmic expression of TH, either in the autopsied PLAN or PD brains studied here, although this occurs early in PD [21]. This suggests that the presence of LB would not necessarily convey negative effects on the micro-environment of neurons, in spite of the neurotoxic materials inside the LB.

A certain proportion of LB-bearing neurons is reported to occur in the substantia nigra (3.6 % on average), and this does not correlate with the symptoms or disease duration [14, 18]. In our case of PLAN, a high proportion of dopaminergic neurons harbor LBs, suggesting the long-term survival of these neurons. In particular, the neurons were preserved well in the locus coeruleus, despite the presence of multiple LBs. This suggests that LBs in PLAN have a low toxicity. As αSyn/PαSyn-loading mitochondria with degenerated inner membrane did not lead to neuronal death in mice, high concentrations of mitochondrial membrane components on the surface of LBs may be associated with the low toxicity of LBs in PLAN through the stabilization of the mitochondrial lipid membrane [7, 29].

**Fig. 8:**
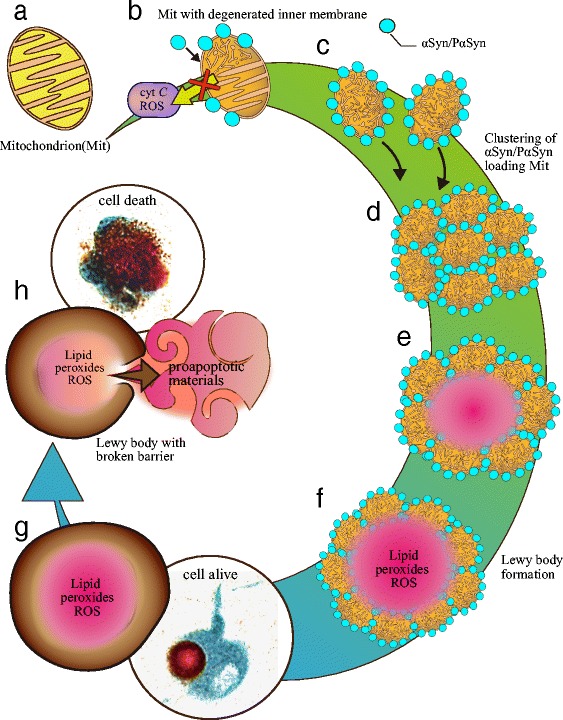
The hypothetical scheme of the process of Lewy body formation in *PLA2G6*-associated neurodegeneration (PLAN). **a** Normal mitochondrion. **b** αSyn/PαSyn accumulates at the damaged mitochondrial membrane with PLA2G6 dysfunction. **c** High expression of αSyn/PαSyn stabilizes the mitochondrial membrane. **d** Due to the high affinity of αSyn for damaged mitochondrial membranes, mitochondria loaded with αSyn/PαSyn are clustered. **e** The cluster of the damaged mitochondria forms a small inclusion that contains toxic materials inside. **f** The small inclusion becomes large to be a Lewy body (LB), which boundary is formed by αSyn/PαSyn and mitochondrial membrane components. **g** When the boundary, which is sufficiently strong to sequester toxic materials inside, the LB-bearing neuron survives for a long time. **h** When this boundary breaks down, pro-apoptotic materials are released into the neuron, leading to rapid neuronal death. The image of neurons double-stained with immunohistochemistry for PαSyn (in brown) and tyrosine hydroxylase (in blue) is taken from the locus coeruleus of a patient with PLAN in (**g**), and a patient with PD in (**h**)

We illustrate this hypothesis in Fig. 8. Because of *PLA2G6* dysfunction, the mitochondrial inner membrane is damaged early on. Strong affinity of αSyn/PαSyn for damaged mitochondrial membrane induces Syn/PαSyn to accumulate on the membrane. Elevated expression of αSyn/PαSyn stabilizes the mitochondrial membrane and prevents membrane permeabilization. The strong affinity of αSyn for damaged mitochondrial membranes also causes clustering of the damaged mitochondria to form LB. The outer structure, which is comprised of a mitochondrial membrane component, functions as a barrier and allows LB-bearing neurons to survive for a long time. When the barrier breaks down, pro-apoptotic materials, which are elevated in PLA2G6 deficiency [20], are released into the cytoplasm, followed by rapid cell death.

In conclusion, αSyn/PαSyn accumulation in damaged mitochondria of *Pla2g6*-KO mice and LBs in PLAN indicates a common pathological mechanism, which includes the strong affinity of αSyn for damaged mitochondrial membranes. PLAN patients with late onset tend to show prominent αSyn accumulation in neurons [31]. Endogenous αSyn could be neuroprotective by stabilizing mitochondrial membranes in neurodegenerative diseases, such as PLAN and possibly even in PD, which may indicate a new target for the treatment of α-synucleinopathy.
